# Analysis of clinical risk factors of premature infants in Chinese women based on anemia status

**DOI:** 10.3389/fped.2025.1690771

**Published:** 2025-11-13

**Authors:** Weiwei Ou, Zhu Xu, Dan Liu

**Affiliations:** Zhongjiang Maternal and Child Health-Care Hospital, Deyang, Sichuan, China,

**Keywords:** preterm infant, anemia, clinical high-risk, fetal dysplasia, placental abruption, severe anemia

## Abstract

**Background:**

Anemia in premature infants, a high-risk category of patients, has been shown to impose significant economic and psychological burdens on families and society at large. Anemia is the most prevalent disease among pregnant women. The impact of anemia on the clinical high-risk status of premature infants remains elucidated; therefore, this study aims to investigate the risk factor of clinical high-risk infants in Chinese women based on anemia status.

**Method:**

A retrospective analysis of the data from premature infants in four medical centres was conducted from January 2023–May 2025. The data, including demographic information, medical histories, gestational diseases, fetal development, and fetal position, were collected. The analysis to identify the factors contributing to the clinical high risk of premature infants was conducted between the anemia groups.

**Results:**

A total of 191 subjects were involved, with an average age of 29.20 ± 5.00 years, but only 34 (17.8%) cases were classified as clinical high-risk. The mean weight of the infants was recorded as 2483 ± 458 g, and the mean gestational age was determined to be 34.96 ± 1.20 weeks, including 72(37.7%) females. A statistically significant variation was observed among the anemia groups concerning maternal age, hypertension, uterine abnormalities, scarred uterus, and placental abnormalities (*p* < 0.05). However, no statistically significant difference was found between high-risk and low-risk premature infants (*p* = 0.838). In the nonanemia group, a statistically significant difference was observed among the variables of gender, gestational hypertension, placental abnormalities, placental abruption, umbilical cord abnormalities, and fetal dysplasia (*p* < 0.05), with male was the protective factor [*OR* = 0.240, 95% CI = (0.076, 0.764)], while placental abruption [*OR* = 31.499, 95% CI = (2.707, 366.599)], and fetal dysplasia [*OR* = 16.927, 95% CI = (3.161, 90.630)] were risk factor. In the anemia group, mild anemia, severe anemia, and placenta previa were found to be statistically significant (*p* < 0.05), but only severe anemia was a high-risk factor [*OR* = 18.600, 95% CI = (1.757, 196.927)].

**Conclusion:**

The findings of this study demonstrate that anemia exerts a significantly different influence on the clinical high-risk symptoms of premature infants. These differences can provide important reference points for managing pregnant women.

## Introduction

Premature infants are defined as newborns born before 37 weeks of gestation. Current estimates indicate that the number of premature infants worldwide may reach 130 million by 2025, constituting approximately one-tenth of all live births (1). Moreover, the birth rate of premature infants in China is approximately 5% (2), and there has been no significant change in recent years (3). The incidence of premature infants varies geographically within China, which is higher in the regions with more developed economies (4) and is gradually increasing (2). These infants exhibit reduced intelligence (5) and low-risk behaviour (6), which adversely impact their academic performance during adolescence (7) and the establishment of social connections in adulthood (8). Premature infants are also accompanied by a high incidence of respiratory, gastrointestinal, endocrine, neurodevelopmental, and other systemic diseases (9). Furthermore, a negative correlation has been demonstrated between the incidence of premature birth and the level of economic development, with the highest rates in sub-Saharan Africa and South Asia (1). Moreover, premature birth was associated with increased medical expenditures and readmission rates (10), which was a significant contributing factor to the mortality rate of children under the age of five (11). Consequently, the prevalence of premature birth and premature infants has been demonstrated to impose a significant burden on families and society at large (12).

Anemia during pregnancy constitutes a significant global problem. Iron deficiency anemia (IDA) is considered to be the most common nutritional deficiency worldwide, affecting approximately 30% of the global population (13). Anemia during pregnancy has been associated with many adverse consequences for both mothers and infants, including an increased risk of premature birth, stillbirth, and neurocognitive impairment (14). The prevalence of anemia among pregnant women in China has been documented as high as 23.5% (15), which has been associated with adverse outcomes, including reduced birth weight (16), premature birth (17), and even postpartum hemorrhage due to tension loss (18), resulting in a considerable threat to the health of pregnant women in China. The prevalence of anemia among pregnant women in Northwest China has been documented as high as 65.1% (19), which may be attributed to the imbalanced economic development and cultural variations (20). The implementation of the national multiple birth policy has been associated with a consistent upward trend in the incidence of premature infants across various geographical regions (21). Consequently, the objective of this study is to investigate the correlation between anemia in pregnant women and the clinical high risk of premature infants through retrospective research, with the aim of providing novel insights for the protection of the health of regional pregnant women and newborns.

## Methods and materials

### Patients

Preterm infants were defined as infants with gestational age <37 weeks, followed by Work Standards for Premature Infants’ Healthcare published by the National Health Commission of the People's Republic of China (https://www.nhc.gov.cn/fys/c100078/201703/bd1c0e0d58644bf3b234150cdf5cb4ca.shtml). Infants with a birthweight of less than 2,000 grams or a gestational age of less than 34 weeks were designated as the clinical high-risk group, while the remaining preterm infants were classified as the clinical low-risk group. A total of 384 premature infants and their mothers were collected from four medical institutions from January 2023 to May 2025. The study was reviewed and endorsed by the ethics committee of Zhongjiang Maternal and Child Health-Care Hospital (ID: 2024ky-001), and all subjects provided written consent in paper or electronic format. This report followed the Strengthening the Reporting of Observational Studies in Epidemiology (STROBE) Statement: guidelines for reporting observational studies (22), as shown in the Supplementary Appendix A1.

### Data collection

**Figure 1:**
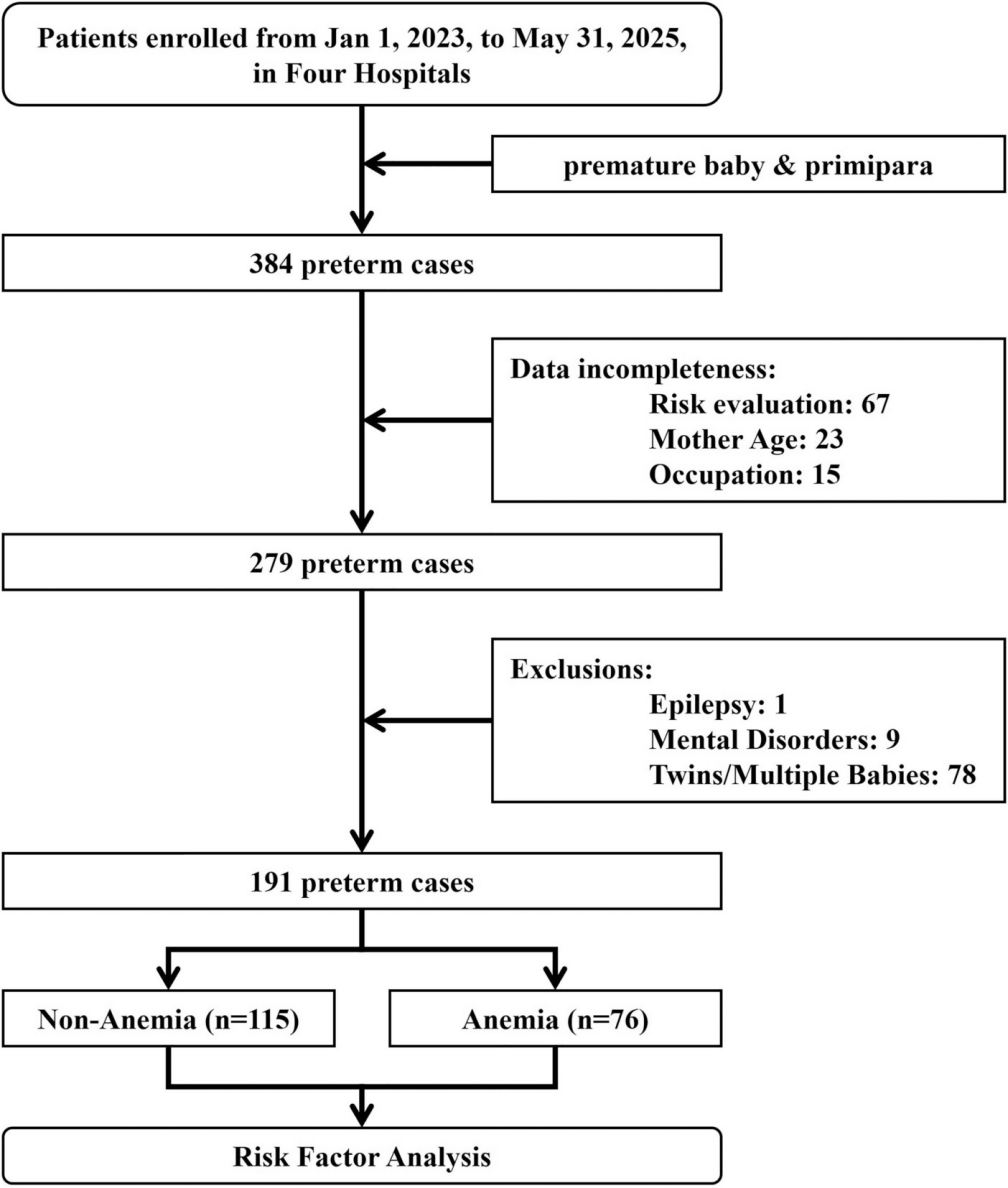
The screening procedure for collecting cases.

Before the data collection procedure, Weiwei Ou and Zhu Xu were trained in the standardization of data collection (*kappa* = 0.823). A comprehensive data set was compiled, encompassing the demographic characteristics of subjects, including age, educational attainment, occupational status, and family history. Additional factors contributing to premature birth, such as diabetes, hypertension, hepatitis B, and pregnancy complications (e.g., eclampsia, diabetes, anemia, etc.), were also considered. The study further examined threatened abortion, placenta previa, placental abruption, abnormal fetal membranes, and infection as potential complicating factors. The infant's information is relevant for consideration: gender, birth weight, abnormal development (abnormal umbilical cord, abnormal fetal position, etc.), and other clinical indications. The subjects diagnosed with epilepsy, genetic diseases, twins/multiple births, or incomplete data were excluded. Following the screening process as shown in Figure 1, 191 cases were selected for data analysis. According to the World Health Organization (WHO), anemia is defined as a hemoglobin concentration below 110 g/L in any trimester of pregnancy (23), with mild anemia (100–109 g/L), moderate anemia (70–99 g/L) and severe anemia (<70 g/L).

### Statistical analysis

SPSS 26 (IBM Corporation, New Orchard Road, Armonk, NY 10,504, USA) was used for statistics. Intra-group differences were compared by *t*-test, 2-sample Kolmogorov–Smirnov test, *χ*^2^ or Fisher's exact test. Binary logistic regression was used for independent risk factor analysis of high-risk neonates. To address the issue of multiple comparisons, the False Discovery Rate (FDR) was implemented to adjust for multiple comparisons. Continuous variables were shown as mean ± standard deviation ($\bar{x}\pm \mathrm{std}.$). A *p*-value of less than 0.05 was considered statistically significant.

## Results

### Patients

The mean age of the 191 mothers was 29.2 ± 5.0 years old, 76 (39.8%) of whom had anemia, 36 (18.8%) received a higher education, and 11 (5.8%) had a family history. The sample included 34(17.8%) clinical high-risk premature infants, with a weight of 2,483 ± 458 g, aged 35.0 ± 1.3 weeks, and 72 (37.7%) female infants.

A subsequent analysis revealed that there were statistically significant differences between the age of the mother, the presence of hypertension, gestational diabetes, uterine abnormalities, scarred uterus, and placental abnormalities between the anemia group and the nonanemia group (*p* < 0.05), as demonstrated in Table 1, but the age of mother (*p* = 0.016), scarred uterus (*p* < 0.001), hypertension (*p* = 0.013) and placenta previa (*p* = 0.013) passed the FDR correction. Additionally, the analysis reveals that there is no statistically significant difference between premature infants in the clinical high-risk and low-risk groups [*OR* = 0.924, 95% CI = (0.431, 1.978), *p* = 0.838].

**Table 1:** The basic information of the participants included is based on anemia (*n* = 191).

Variables	Group	Anemia^a^			
		No ($\bar{x}\pm d$, *n*)	Yes ($\bar{x}\pm d$, *n*)	*t*, *χ*^2^	*P*
Mother					
Age (Years)		30.0 ± 5.2	27.9 ± 4.5	2.980	0.003
Higher education	No	93	62	0.015	0.902
	Yes	22	14		
Family History	No	109	71	0.156	0.693
	Yes	6	5		
Job	No	18	9	0.880	0.348
	Freelancer	58	37		
	Yes	39	30		
Thalassemia	No	115	68		<0.001
	Yes	0	8		
Mild Anemia (100–109 g/L)	No	115	42	62.589	<0.001
	Yes	0	34		
Moderate Anemia (70–99 g/L)	No	115	41	64.843	<0.001
	Yes	0	35		
Severe Anemia (<70 g/L)	No	115	72		0.024
	Yes	0	4		
Disease					
Diabetes	No	85	48	2.503	0.114
	Yes	30	28		
Hypertension	No	101	76	9.984	0.002
	Yes	14	0		
Obesity	No	111	70		0.200
	Yes	4	6		
Hypoproteinemia	No	115	74		0.157
	Yes	0	2		
Hyperlipidemia	No	113	70		0.061
	Yes	2	6		
Hepatitis B	No	97	71	3.557	0.059
	Yes	18	5		
Hypothyroidism	No	100	65	0.080	0.778
	Yes	15	11		
Intrahepatic Cholestasis	No	107	65	2.886	0.089
	Yes	8	11		
Gestational Complications					
Gestational Diabetes	No	90	47	6.083	0.014
	Yes	25	29		
Gestational Hypertension	No	105	74	2.858	0.091
	Yes	10	2		
Gestational Infection	No	91	61	0.036	0.849
	Yes	24	15		
Respiratory Tract Infection	No	95	65	0.286	0.592
	Yes	20	11		
Gestational Other Disease	No	109	72		0.999
	Yes	6	4		
Blood Type Controversy	No	114	75		0.999
	Yes	1	1		
Amniotic Fluid Anomaly	No	113	71		0.117
	Yes	2	5		
Uterine Anomaly	No	103	60	4.124	0.042
	Yes	12	16		
Scarred Uterine	No	99	45	17.817	<0.001
	Yes	16	31		
Placenta Previa	No	103	55	9.468	0.002
	Yes	12	21		
Abruptio Placentae	No	110	67	3.784	0.052
	Yes	5	9		
Oviduct Anomaly	No	111	74		0.999
	Yes	4	2		
IVF-ET	No	115	74		0.157
	Yes	0	2		
Fetus					
Clinical Risk	Low	94	63	0.042	0.838
	High	21	13		
Gender	Female	39	33	11.761	0.184
	Male	76	43		
Weight (g)		2,472 ± 464	2,500 ± 450	−0.416	0.678
Gestation (Week)		34.94 ± 1.37	35.00 ± 1.13	−0.321	0.748
Position Anomaly	No	110	67	3.784	0.052
	Yes	5	9		
Transverse Presentation	No	113	74		0.651
	Yes	2	2		
Umbilical Cord Anomaly	No	99	65	0.012	0.913
	Yes	16	11		
Development Anomaly	No	107	67	1.347	0.246
	Yes	8	9		
Membrance Rupture	No	86	48	2.953	0.860
	Yes	29	28		

IVF-ET, *In vitro* fertilization and embryo transfer. All *p* values were corrected by false discovery rate.

a Hemoglobin concentration below 110 g/L during pregnancy is considered anemia.

### Risk factor analysis

**Nonanemia group**: Table 2 showed that statistical significances were found in gender, gestational hypertension, placental abnormalities, placental abruption, umbilical cord abnormalities, and fetal development abnormalities (all *p* < 0.05), but only development abnormalities (*p* = 0.037) passed FDR correction. Binary logistic regression analysis demonstrated that men exhibited a protective effect [*OR* = 0.240, 95% CI = (0.076, 0.764)], while placental abruption [*OR* = 31.499, 95% CI = (2.707, 366.599)] and fetal dysplasia [*OR* = 16.927, 95% CI = (3.161, 90.630)] were identified as significant contributors to the risk of severe premature births, as illustrated in Table 3.

**Table 2:** Analysis of related factors for clinical high-risk levels in neonates based on anemia groups.

Variables	Group	Non-Anemia (*n* = 115)				Anemia (*n* = 76)			
		Low Risk ($\bar{x}\pm d$, *n*)	High-Risk ($\bar{x}\pm d$, *n*)	*t, χ* ^2^	*P*	Low-Risk ($\bar{x}\pm d$, *n*)	High-Risk ($\bar{x}\pm d$, *n*)	*t, χ* ^2^	*P*
Mother									
Gestational Anemia	No	94	21		NA	42	8		0.787
	Yes	0	0			21	5		
Thalassemia	No	94	21		NA	57	10		0.178
	Yes	0	0			6	3		
Mild Anemia (100–109 g/L)	No	94	21		NA	31	11	5.465	0.019
	Yes	0	0			32	2		
Moderate Anemia (70–99 g/L)	No	94	21		NA	35	6	0.383	0.536
	Yes	0	0			28	7		
Severe Anemia (<70 g/L)	No	94	21		NA	62	10		0.015
	Yes	0	0			1	3		
Age		30.2 ± 5.1	29.4 ± 5.3	0.641	0.523	27.7 ± 4.5	28.9 ± 4.4	−0.871	0.387
Higher Education	No	78	15		0.231	51	11		0.999
	Yes	16	6			12	2		
Family History	No	89	20		0.999	59	12		0.999
	Yes	5	1			4	1		
Job	No	16	2	0.848	0.654	8	1	0.318	0.853
	Freelancer	46	12			30	7		
	Yes	32	7			25	5		
Disease									
Diabetes	No	68	17	0.660	0.416	38	10		0.351
	Yes	26	4			25	3		
Hypertension	No	84	17		0.283	63	13		NA
	Yes	10	4			0	0		
Obesity	No	91	20		0.559	57	13		0.582
	Yes	3	1			6	0		
Hypoproteinemia	No	94	21		NA	61	13		0.999
	Yes	0	0			2	0		
Hyperlipidemia	No	93	20		0.333	58	12		0.999
	Yes	1	1			5	1		
Hepatitis B	No	77	20		0.188	60	11		0.200
	Yes	17	1			3	2		
Hypothyroidism	No	84	16		0.146	52	13		0.194
	Yes	10	5			11	0		
Intrahepatic Cholestasis	No	88	19		0.6	52	13		0.194
	Yes	6	2		37	11	0		
Gestational Complications									
Gestational Diabetes	No	73	17		0.999	37	10		0.348
	Yes	21	4			26	3		
Gestational Hypertension	No	89	16		0.017	62	12		0.315
	Yes	5	5			1	1		
Gestational Infection	No	76	15		0.376	49	12		0.444
	Yes	18	6			14	1		
Respiratory Tract Infection	No	78	17		0.759	52	13		0.194
	Yes	16	4			11	0		
Gestational Other Disease	No	89	20		0.999	61	11		0.133
	Yes	5	1			2	2		
Blood Type Controversy	No	93	21		0.999	62	13		0.999
	Yes	1	0			1	0		
Amniotic Fluid Anomaly	No	93	20		0.333	59	12		0.999
	Yes	1	1			4	1		
Uterus Anomaly	No	86	17		0.228	48	12		0.278
	Yes	8	4			15	1		
Scar Uterus	No	80	19		0.733	39	6	1.102	0.293
	Yes	14	2			24	7		
Placenta Anomaly	No	87	16	4.917	0.027	48	7	2.691	0.101
	Yes	7	5			15	6		
Placenta Previa	No	89	21		0.583	58	9		0.041
	Yes	5	0			5	4		
Abruptio Placentae	No	93	18		0.019	62	12		0.315
	Yes	1	3			1	1		
Oviduct Anomaly	No	94	21		NA	61	13		0.999
	Yes	0	0			2	0		
IVF-ET	No	94	21		NA	61	13		0.999
	Yes	0	0			2	0		
Fetus									
Gender	Female	28	11	3.910	0.048	26	7	0.694	0.405
	Male	66	10			37	6		
Position Anomaly	No	89	21		0.583	55	12		0.999
	Yes	5	0			8	1		
Transverse Presentation	No	92	21		0.999	62	12		0.315
	Yes	2	0			1	1		
Umbilical Cord Anomaly	No	84	15	4.609	0.032	54	11	0.011	0.918
	Yes	10	6			9	2		
Development Anomaly	No	91	16	12.274	0.001	56	11	0.189	0.664
	Yes	3	5			7	2		
Membrance Rupture	No	70	16	0.027	0.869	37	11	3.103	0.078
	Yes	24	5			26	2		

IVF-ET, *In vitro* fertilization and embryo transfer; NA, not available.

**Table 3:** The binary logistic analysis of the risk factor of the neonates is based on anemia.

Subgroup	Wald χ^2^	P	Exp(B)	95% CI		R^2^	AUC
				Lower	Upper		
Non-Anemia							
Male	5.836	0.016	0.240	0.076	0.764	0.289	0.763
Abruptio Placentae (Yes)	7.591	0.006	31.499	2.707	366.559		
Fetal Development Anomaly (Yes)	10.92	0.001	16.927	3.161	90.630		
Anemia							
Severe Anemia (Yes)	5.895	0.015	18.600	1.757	196.927	0.147	0.607

AUC, area under curve.

**Anemia group**: As illustrated in Table 2, there were statistically significant differences found in mild anemia, severe anemia, and placenta previa (all *p* < 0.05), but none passed FDR correction. Only severe anemia [*OR* = 18.600, 95% CI = (1.757, 196.927)] was the risk factor for clinical high-risk in preterm neonates, as illustrated in Table 3.

## Discussion

Despite the absence of a statistically significant discrepancy in the prevalence of anemia between premature infants classified as high-risk and low-risk infants, a discernible divergence in the contributing factors emerged when comparing the anemia group to the nonanemia group. The former exhibited a preponderance of female infants, placental abruption, and fetal dysplasia. In contrast, the latter demonstrated a notable absence of severe anemia. These observations underscore the potential for targeted clinical management interventions and health guidance for expectant mothers, thereby enhancing maternal and fetal well-being.

### Female and clinical high-risk

Fetal gender differences may also be a factor leading to the risk of premature birth (24). Pregnant women carrying female fetuses may be susceptible to premature delivery due to other complications, such as hypothyroidism (25). Furthermore, female fetuses demonstrate a heightened susceptibility to the impact of ambient temperature, particularly in instances of extreme temperature, which can result in preterm birth (26). For example, female fetuses in Germany are more susceptible to heat stress during pregnancy, and extremely high temperatures and long-term high temperature exposure increase the risk of premature birth, which may be due to the change of vascular resistance in the uterine artery caused by heat exposure (27). However, research indicates that pregnant women in Western and Northern China exhibit heightened sensitivity to the cold wave; in particular, pregnant women carrying female fetuses may experience an elevated risk of premature birth when exposed to the cold wave (28). Consequently, the clinical management of fetuses may be contingent on gender, with mothers carrying female fetuses being advised to focus on the external factors, particularly temperature.

### Placental abruption and clinical high-risk

Placental abruption is defined as the premature separation of the placenta and uterine attachments prior to fetal delivery (29), which may increase the risk of premature delivery, severe maternal complications, and adverse neonatal outcomes (30). Placental abruption has been associated with several potential etiologies, including thrombosis, inflammation, infection, and uterine placental vascular lesions (31), leading to a range of chronic pathological changes gradually, such as placental perfusion insufficiency, placental infarction, and spiral artery remodelling defects (32). It is important to note that these defects may, in turn, contribute to the development of placental abruption. Consequently, premature births accompanied by lesions may manifest symptoms such as vaginal bleeding (33) and discomfort. We recommend that a meticulous blood and ultrasound examination be conducted for confirmation in the early clinical suspicion (34). Furthermore, the substantial mechanical force and shear force on the abdomen may result in placental abruption (35). This condition can be further influenced by maternal factors, including age >35, race, low BMI, smoking during pregnancy, and previous placental abruption history (36). Additional contributing factors include placenta previa, pregnancy-induced hypertension, and other elements. Consequently, in clinical practice, it is imperative to promptly assess pregnant women experiencing abdominal pain, vaginal bleeding, and abnormal fetal heart rate patterns, intending to enhance the management of maternal high-risk factors, and aiming at mitigating the risk of premature birth attributable to placental abruption.

### Fetal dysplasia and clinical high-risk

In the present study, fetal dysplasia was combined with a variety of other anomalies, including fetal distress, fetal growth restriction, fetal tricuspid regurgitation, and fetal permanent right umbilical vein. Fetal distress is a symptom of fetal intrauterine hypoxia, which may be manifested by umbilical artery and cerebral artery abnormalities (37). For instance, umbilical artery thrombosis has been associated with premature birth (38) and a significant escalation in the risk of refeeding syndrome in premature infants (39). Maternal exposure to air pollution (40) and fetal umbilical cord abnormalities (41) have been demonstrated to increase the risk of fetal distress significantly. Fortunately, a variety of diagnostic procedures, including fetal heart rate monitoring, ultrasound arterial examination, and meconium-stained amniotic fluid assessment, can be utilized to evaluate the risk of fetal distress in a clinical setting. Therefore, a comprehensive review of the patient's medical history is imperative to ascertain the risk of fetal development distress. This requires a meticulous monitoring regimen during pregnancy to mitigate the potential complications.

Fetal growth restriction (FGR) may be attributed to chronic fetal hypoxia resulting from inadequate placental perfusion (42). Abnormal fetal metabolism or malnutrition may also lead to FGR (43). Negative emotions of mother may have the potential to disrupt normal metabolic pathways (44), which can have adverse effects on the cardiovascular health of fetus (45) and resulting in FGR and preterm. Fortunately, the abnormal characteristics of premature infants’ growth restriction performance can be identified by Doppler ultrasound (46), and the risk of fetal growth restriction may be reduced by supplementing 50–150 mg of aspirin daily before 16 weeks of gestation (47). Consequently, within the framework of clinical management, it is recommended that fetal growth restriction be regarded as a pivotal monitoring index and management objective during routine examinations.

Although the incidence of other fetal developmental abnormalities, such as tricuspid regurgitation and fetal persistent right umbilical vein, is low, they nevertheless require consideration in the clinical management of pregnant women. Fetal tricuspid regurgitation has been associated with chromosome defects, including Down syndrome (48), which has been linked to an increased risk of coronary heart disease (49). However, ultrasound examination findings indicate that patients with mild fetal tricuspid regurgitation or without structural abnormalities in the early stage of pregnancy are less likely to suffer from severe congenital heart disease after delivery (50). Therefore, accurate identification of tricuspid regurgitation in clinical management is imperative to mitigate the potential anxiety experienced by pregnant women. The prevalence of fetal persistent right umbilical vein (PRUV) is approximately 0.17%, a condition that can be identified through prenatal echocardiography. Although the prognosis for isolated PRUV is favorable, patients with severe PRUV symptoms and structural abnormalities may develop heart-related diseases, thereby increasing the likelihood of premature birth (51). Consequently, it is imperative to allocate clinical attention to these diseases. However, the question of whether these diseases result in the manifestation of high-risk clinical symptoms associated with premature birth requires further investigation.

### Severe anemia

Our research indicates that severe anemia is a crucial factor in the development of adverse clinical manifestations in preterm infants with anemia. Global data show that the prevalence of anemia among pregnant women is 36.8% (458,067/1,244,747) (52), which is consistent with our study's findings (39.8% = 76/191) (*P* = 0.391). The majority of the patients are mildly anemic. However, severe anemia during pregnancy can lead to a decrease in blood volume and oxygen-carrying capacity, potentially resulting in postpartum hemorrhage, premature rupture of the membranes, preterm delivery, low birth weight, cesarean section, and neonatal respiratory distress syndrome (53). Research indicates that Chinese anemia in pregnant women may be associated with nutritional deficiencies, regional differences, dietary habits, economic status (54), and genetics (55). The severity of anemia is directly related to the risk of preterm delivery (56). Although meta-analyses have not demonstrated a substantial clinical benefit from antenatal nutritional supplementation (57), they have been shown to improve the outcomes of other pregnancy complications (58). Therefore, it is essential for healthcare providers to thoroughly investigate the underlying causes of anemia in pregnant women and to implement appropriate nutritional interventions to improve the outcomes for both the mother and the fetus.

### Limitation

Despite conducting a multicenter retrospective study, the limited sample size precluded the execution of a comprehensive hierarchical analysis of gender or anemia types, including thalassemia and iron deficiency anemia. This discrepancy can be attributed, at least in part, to the study's primary objective, which was to ascertain whether premature infants are clinically high-risk. The number of infants exhibiting high-risk clinical symptoms is minimal, with only 34 cases identified. This may potentially lead to an inadequate statistical efficacy in the subgroup analysis of anemia (i.e., 4 severe anemia cases, with only 3 in high-risk group). Furthermore, cases of lower weight (i.e., <2,000 g) or gestational weeks (i.e., <34) may have implications for the sensitivity analysis focused on the anemia group. Secondly, the decline in fertility in China in recent years has also led to a decrease in the number of premature infants (59). Thirdly, we excluded twins from our sample because the identical maternal environment in twins may result in inconsistent premature infant statuses. Furthermore, the weight of each twin will be significantly lower than that of a non-twin (2,211 ± 423 g vs. 2,483 ± 457 g, *p* < 0.001). The exclusion of these variables from the study design may have resulted in significant alterations to the study's findings. A fourth limitation of our study is that it did not include data on minority nationality, living habits, dietary habits, family economic level, anemia gene screening results, or father's disease history (54, 55). These factors may have impacted the results. Consequently, this study constitutes a preliminary exploration of the relationship between anemia and clinical risk factors in premature infants, thereby establishing a foundation for more in-depth research.

## Conclusion

Anemia, as a prevalent clinical ailment among pregnant women, poses a significant threat to the health of both the mother and the fetus, particularly in cases of premature infants. The findings of this study demonstrate that anemia exerts a significantly different influence on the clinical high-risk symptoms of premature infants. These differences can provide important reference points for the individual clinical management of pregnant women.

## Data Availability

The raw data supporting the conclusions of this article will be made available by the authors, without undue reservation.
