# Supplementary material for: Analysis of clinical risk factors of premature infants in Chinese women based on anemia status
Source: Front Pediatr. 2025 Nov 13;13:1690771. doi: 10.3389/fped.2025.1690771 (PMC12657454; doi:10.3389/fped.2025.1690771)
Supplement: Supplementary file 1 [file Datasheet1.pdf]

Appendix A1. STROBE Checklist

	Item No	Recommendation	Page No
**Title and abstract**	1	(*a*) Indicate the study's design with a commonly used term in the title or the abstract	Page 1
		(*b*) Provide in the abstract an informative and balanced summary of what was done and what was found	Page 1
**Introduction**			
Background/rationale	2	Explain the scientific background and rationale for the investigation being reported	Page 2
Objectives	3	State specific objectives, including any prespecified hypotheses	Page 2, Lines 161-166-60
**Methods**			
Study design	4	Present key elements of study design early in the paper	Page 2-3, the Method and Materials section
Setting	5	Describe the setting, locations, and relevant dates, including periods of recruitment, exposure, follow-up, and data collection	Page 2, patients
Participants	6	(*a*) Give the eligibility criteria, and the sources and methods of selection of participants	Page 2, Lines 172-180
Variables	7	Clearly define all outcomes, exposures, predictors, potential confounders, and effect modifiers. Give diagnostic criteria, if applicable	Page 2, lines 197-210
Data sources/ measurement	8*	For each variable of interest, give sources of data and details of methods of assessment (measurement). Describe comparability of assessment methods if there is more than one group	Page 2, lines 197-210
Bias	9	Describe any efforts to address potential sources of bias	Page 2-3, statistics
Study size	10	Explain how the study size was arrived at	Figure 1,
Quantitative variables	11	Explain how quantitative variables were handled in the analyses. If applicable, describe which groupings were chosen and why	Page 2-3, statistics
Statistical methods	12	(*a*) Describe all statistical methods, including those used to control for confounding	Page 2-3, statistics
		(*b*) Describe any methods used to examine subgroups and interactions	Page 2-3, statistics
		(*c*) Explain how missing data were addressed	Not Available
		(*d*) If applicable, describe analytical methods taking account of sampling strategy	Not Available
		(*e*) Describe any sensitivity analyses	Not Available
**Results**			
Participants	13*	(a) Report numbers of individuals at each stage of study—eg numbers potentially eligible, examined for	Table 1 and Table 2

		eligibility, confirmed eligible, included in the study, completing follow-up, and analysed	
		(b) Give reasons for non-participation at each stage	Not Available
		(c) Consider use of a flow diagram	Figure 1
Descriptive data	14*	(a) Give characteristics of study participants (eg demographic, clinical, social) and information on exposures and potential confounders	Table 1
		(b) Indicate number of participants with missing data for each variable of interest	Not Available
Outcome data	15*	Report numbers of outcome events or summary measures	Page 3, lines 276-328, Table 1
Main results	16	(*a*) Give unadjusted estimates and, if applicable, confounder-adjusted estimates and their precision (eg, 95% confidence interval). Make clear which confounders were adjusted for and why they were included	Page 3-5, lines 334-488,<br>Table 1, and Table 2, Table 3.
		(*b*) Report category boundaries when continuous variables were categorized	Anaemia level defined in Table 1 and Table 2
		(*c*) If relevant, consider translating estimates of relative risk into absolute risk for a meaningful time period	Not Available
Other analyses	17	Report other analyses done—eg analyses of subgroups and interactions, and sensitivity analyses	Page 3-5, lines 334-488
**Discussion**			
Key results	18	Summarise key results with reference to study objectives	Page5, lines 494-503
Limitations	19	Discuss limitations of the study, taking into account sources of potential bias or imprecision. Discuss both direction and magnitude of any potential bias	Page 8-9, lines 871-899
Interpretation	20	Give a cautious overall interpretation of results considering objectives, limitations, multiplicity of analyses, results from similar studies, and other relevant evidence	Page 5-8, lines 533-550, 558-771, 782-797, 802-813, 817-836, 843-861
Generalisability	21	Discuss the generalisability (external validity) of the study results	Page 5-8, lines 550-553, 771-777, 797-801, 813-816, 836-840, 861-865
**Other information**			
Funding	22	Give the source of funding and the role of the funders for the present study and, if applicable, for the original study on which the present article is based	Page 9, Funding

*Give information separately for exposed and unexposed groups.

Note: An Explanation and Elaboration article discusses each checklist item and gives methodological background and published examples of transparent reporting. The STROBE checklist is best used in conjunction with this article (freely available on the Web sites of PLoS Medicine at http://www.plosmedicine.org/, Annals of Internal Medicine at http://www.annals.org/, and Epidemiology at http://www.epidem.com/). Information on the STROBE Initiative is available at www.strobe-statement.org.
